# The Impact of Forced Separations Between Women and Their Pets in Domestic Violence Situations and the Effectiveness of Crisis Response: Protocol for a Conceptual Framework

**DOI:** 10.2196/52067

**Published:** 2024-01-25

**Authors:** Jasmine Montgomery, Janice Lloyd, Zhanming Liang

**Affiliations:** 1 College of Public Health, Medical and Veterinary Sciences James Cook University Queensland Australia

**Keywords:** companion animal, domestic violence, forced separation, research protocol, animal welfare, pets, animal abuse, Australia, coercive control, victim, abusive partner, abusive, women, trauma, support, animal

## Abstract

**Background:**

Women are at high risk of experiencing trauma, guilt, and stress when forced to separate from their companion animals when fleeing domestic violence. Where little support is available for women and pets to stay together, women may be forced to delay leaving the abusive relationship or leave the pet with the abuser. Forced separation places both women and pets at substantial risk, where pets may be used as a coercive control measure. However, little evidence exists regarding the extent to which Australian services or policies offer support in these circumstances.

**Objective:**

This research aims to increase the understanding and the impacts of forced separation between women and their pets in domestic violence situations. The research will investigate the effectiveness of service responses for both women and animals, aiming to develop a policy framework that guides service improvement with the goal of enhancing outcomes for women and pets fleeing domestic violence.

**Methods:**

This protocol paper describes the process of developing a conceptual framework of 4 studies that include a scoping review, policy analysis, focus groups, and interviews that guide the design of the qualitative research project.

**Results:**

A scoping review of the literature on forced separation from pets in domestic violence, natural disasters, and homelessness situations has led to the development of a conceptual framework that guided the design of the proposed study. The review also confirmed the necessity of the proposed research project in addressing the lack of Australian national frameworks and guidance available for women and pets seeking formal support in domestic violence situations. As of August 2023, supporting organizations have commenced the distribution of the research flyers. Expected data collection will be completed between August and October 2023. The results are expected to be published in June 2025.

**Conclusions:**

Via a systematic process, the importance of the proposed study in improving the understanding of the impact of forced separation between women and their pets at times of domestic violence and the gaps in best supporting both women and their pets has been confirmed. A study design based on the learnings from previous studies and the focus of the current research has been finalized. The impact of the research project in developing an Australian national framework for best supporting women and their pets in crisis situations is anticipated.

**International Registered Report Identifier (IRRID):**

PRR1-10.2196/52067

## Introduction

In Australia, 69% of households live with a companion animal (pet), and 86% of households with a pet have children [1]. Dogs and cats are the most popular type of pets [1]. The main reason for living with a companion animal is companionship [1]. The relationship is considered beneficial both psychologically and physiologically for humans and animals [2]. Pets are a vital support system providing emotional support or strength at times of domestic violence [3]. Survivors in domestic and family violence situations often live in terror and face threats to themselves and their pets [4]. Sadly, women in domestic violence situations are often faced with the torturous decision to leave their pet with the perpetrator to seek safety or access temporary fostering, resulting in forced separation from their companion animal (Montgomery et al [5], in press), thus losing the emotional support normally received from the relationship [3].

Barrett et al [6] found that decisions to leave or stay in the relationship were impacted by the concerns for the animal’s welfare, with 56% of women delaying leaving the relationship to protect their pet. Women with both children and pets were also found to delay leaving an abusive relationship out of concern for the pet’s welfare [7]. Most women who delayed were forced to leave their companion animal with the perpetrator when they eventually fled to safety and 47% of women would have fled to safety with their companion animal if support was available [8]. Completing a safety plan when leaving domestic violence situations was often compromised due to a lack of pet-inclusive shelters, often leading to homelessness in order to stay with their pet [7]. When survivors are forced to leave their companion animals with the perpetrator, the risk of coercive control (such as monitoring a person’s movements) increases where the companion animal is used as a coercive control tool [9]. The companion animal in this situation may be subject to continued maltreatment [9], often resulting in torture or death [10] and survivors experience additional guilt and trauma [3] as a result. Often, they consider returning to their partner for the sake of their companion animals’ safety [8]. Where companion animals have survived domestic violence, signs of distress in the animal have been observed through behavioral changes, such as avoidance and vocalization [4,11]. Devastatingly, in Australia, such behavioral changes often result in euthanasia of the pet [4,11].

The emotional attachment between survivors of domestic violence and their pets may be substantial due to sharing the experience of abuse [4], which makes a deliberate act of cruelty or death of a companion animal particularly torturous [12]. While it is the case that domestic violence is a human issue that affects both men and women, it is recognized as a gender-based issue where men are more likely to perpetrate violence against women and is considered an epidemic problem that requires change in Australia [13]. A recent report on homicide in Australia [14] reveals that, from 1989 to 2020, the incidence of intimate partner homicide is consistently much higher for female survivors than male survivors. The most recent statistic (2019-2020) states that female individuals were the targets in 36 (80%) of the 45 intimate partner homicides. Considering Australia is one of the highest pet ownership countries in the world, where women with children are more likely to have a pet [15], it is vital to address the risks for survivors and their companion animals at times of forced separation because of domestic violence. In such a context, a research project has been developed to investigate the existing policy framework and relevant services that provide support to people and companion animals in domestic violence situations. This protocol paper will explain the process of confirming research gaps and determining research questions and will provide details of the overall project design to be used by the proposed project as informed by the learnings from previously published studies.

## Methods

### Overview

A scoping review [5] using the keywords “human-animal relationship/bond,” “pets,” “companion animals,” “animal abuse,” “violence,” “homelessness,” “housing,” and “disasters,” was conducted between March and August 2022. The review focused on identifying empirical studies on the human-animal relationship and crisis or situational change with no date limitation. The review was guided by Arksey and O’Malley’s [16] framework for scoping reviews and conformed to the Preferred Reporting Items for Systematic Reviews and Meta-Analyses (PRISMA) checklist for scoping reviews [17]. English-language, scholarly peer-reviewed papers that included adults with a strong relationship with a pet and an event or change of situation were a criterion for the scoping review. All methodology types were accepted. Gray literature and certain animal types (rodents, wildlife, zoo animals, and working animals) were excluded. The papers were assessed on their ability to fit within the inclusion criteria. Five databases (MEDLINE Ovid, PsycINFO, Scopus, CINAHL, and Emcare Ovid) were searched, and a total of 42 scholarly papers that met the inclusion criteria were identified and included for data extraction. The scoping review mapped the concept of forced separation between people and their companion animals in areas of crisis or situational change and examined policies that included companion animals. The study design and methods used for the studies were also examined to inform the current project design. Please see the full list of papers included in the scoping review in the Multimedia Appendix 1 [3,4,6-11,18-51].

### Scoping Review Findings That Informed the Development of the Protocol

The identified studies in [5] scoping review were predominately quantitative and conducted in the United States, with a focus on the co-occurrence of animal abuse and domestic violence. The lens of research has recently focused on the relationship and animal maltreatment or welfare concerns. Surveys and semistructured interviews were the common forms for collecting quantitative studies and qualitative data respectively. The average sample size consisted of 200 participants for quantitative studies and 20 participants for qualitative studies. The target population was predominantly female adults seeking refuge from domestic violence shelters and support services.

The scoping review [5] confirmed a lack of support for both humans and animals at times of forced separation because of domestic violence. The oversights of the animals’ safety and welfare showed that animals were being left with the abuser [18] and women delayed leaving a violent relationship to protect the companion animal [19]. Additional barriers that were identified included geographical locations, lack of available supports [19], lack of awareness of supports, and attachment or fear of separation from the companion animal [4]. As a result of these barriers, the risks to safety, health, and well-being for women, children, and their companion animals have increased.

The scoping review [5] findings revealed survivors were often reluctant to reach out to services due to a lack of trust in accessing support services, veterinary care, and law enforcement. A lack of trust was associated with a fear of being forced to separate from their companion animal [10,18,20,21]. The reluctance to access support, and the responsibility weighing on women to access supports [6,22] is highly concerning. Although many studies in the literature provided implications for service providers, no research was found that investigated the policy frameworks that provide support to people and companion animals in domestic violence situations at any system, organization, societal, or individual level [5].

### Ethical Considerations

The following ethical considerations are guided by the Global Women’s Institute for the Department of Foreign Affairs and Fair Trade [52], which provides recommendations for projects specific to researching women in domestic violence situations. Ethical approval was granted from the Human Research Ethics Committee (approval H9148). Participation in this study is voluntary and written and verbal consent will be obtained from every participant. The data will be retained for a minimum period of 5 years and will only be accessible to the research team. All data collected will be deidentified and pseudonyms will be provided. All audio recordings for both target populations will be erased after transcription. The primary target population will have the opportunity to review the transcriptions in writing via email. To avoid comprising anonymity and confidentiality for the primary target population, specific locations, age, occupation, culture, and religious discourse in the primary target will not be included in the narrative where there is potential to make the participant identifiable. Consent will be obtained verbally prior to the commencement of the focus group discussion and interviews. Participants are reminded of the voluntary nature of the study and their rights to not answer questions or withdraw their participation from the study. The focus group will be informed that confidentiality is not guaranteed and will be requested to anonymize discussions of their opinions and keep the group discussions private. Confidentiality and anonymity are provided to the interview participants.

### Research Focus and Research Questions

The research aims to inform the Australian policy framework by investigating how support services operate across different contexts for adult women and their companion animals affected by forced separation to reduce negative impacts for both people and animals when fleeing domestic violence situations. The research aim will be achieved by the following two objectives:

1. Identifying the impacts of forced separation between adult female survivors of domestic violence and their companion animal’s health, safety, and living conditions.
2. Identifying the existing strategies and support services, the perceived effectiveness of these strategies, and areas for improvement to develop recommendations that maximize support to people and their companion animals fleeing domestic violence situations.

The research seeks to answer the following two questions.

1. How does forced separation impact the domestic violence survivor and their companion animal under the existing policy and support framework in Australia?
2. What are the factors and how do these factors influence the extent that the benefits of the existing services currently available to people and their companion animals are realized?

A qualitative design will be used to address the gaps in the literature of a lack of national framework to guide pets and women in domestic violence; the impact of forced separation; and the roles, attitudes, and beliefs of seeking and providing services to better understand the impacts and perceptions of forced separation. The transformative paradigm views privilege and power as a social construction that is embedded through social, political, cultural, economic, gender, age, disability, race, and ethnicity. The transformative worldview is a suitable framework providing the lens of power and oppression with a focus on positive social change [53].

### Conceptual Framework

**Figure 1:**
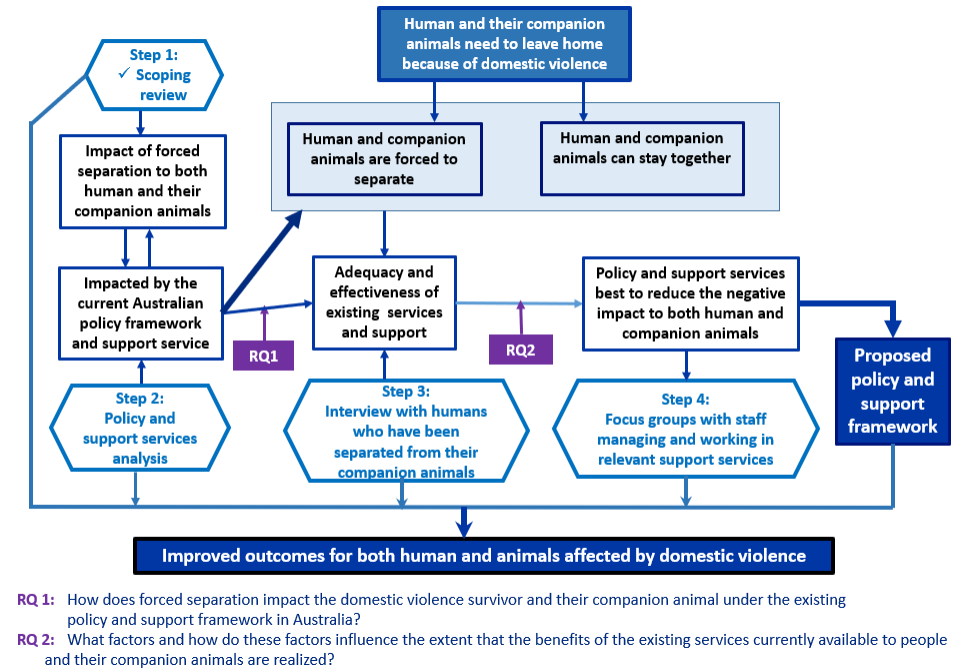
The conceptual framework. RQ: research question.

Based on the findings of the scoping review [5] and the role of support services in preventing or minimizing adverse outcomes due to forced separation, a conceptual framework (Figure 1) was developed. The framework indicates that policy and adequate, effective support services are required to improve the outcomes for people and companion animals who must leave their homes because of domestic violence. The scoping review [5] confirms that a policy framework, key supports, and elements required to achieve these outcomes remain unclear. It is important to understand existing policies, support services or providers, and those who use the services in Australia so that improvements can be made to best support people and companion animals fleeing domestic violence. Guided by the conceptual framework created for this study, 4 steps (Figure 1, studies 1-4) need to be implemented to enrich our understanding of the key elements leading to the development of a policy framework on the forced separation of companion animals because of domestic violence that is relevant to the Australian context. The steps include:

1. A scoping review [5] of forced separation at times of crisis or situation (completed).
2. A policy or services analysis and a scanning of the key supports to humans and animals that will analyze the purpose; construction; implementation; and impacts to understand, evaluate, and provide meaning and context [54].
3. Semistructured individual interviews with participants who have accessed a variety of support services (refuges, crisis services, animal welfare services, and mainstream such as women’s legal services) will be conducted to increase the understanding and impacts of forced separation on people and companion animals.
4. Focus groups with staff and service providers will be conducted to understand and identify perceptions of the effectiveness and adequacy of service provision.

A critical analysis of steps 2-4 will be completed to compare the findings of the most common types of support, service gaps, and availability of services, leading to the development of an improved policy and support framework.

### Project Design and Method

#### Participants: Characteristics and Recruitment

The research project has 2 target populations. The primary target population is female individuals who have or have had considered themselves to have a strong emotional bond in a relationship with their companion animals; have experienced domestic violence; and have been forced to separate from their companion animals or sacrificed their own health, safety, and living arrangements to stay with the animals. The secondary target population is those who have not been directly affected by forced separation and domestic violence but have provided or are currently involved in providing professional support services to the primary target population. Due to the complex and sensitive phenomenon, both target populations are adults, 18 years and older [52].

Both target populations will be recruited through relevant domestic violence or animal welfare organizations. The organizations will be responsible for making direct contact with the potential participants via emails or organization-based advertisements. Potential participants will be encouraged to make direct contact with the principal researcher (first author) should they wish to participate in the study. Both target populations will be geographically recruited nationally across all states and territories in Australia. Due to financial and logistic constraints [55], both target groups are required to be fluent in the English language.

#### Sampling Strategy

Purposive sampling will be used to gather specific characteristics of survivors who have a strong emotional bond with their companion animals (primary target population) to maximize the richness of the data in addressing the research questions [56]. People who received an invitation from their perspective organization and made contact to participate in the study will need to fit the characteristics of either target population 1 or 2.

#### Key Stakeholders

To maximize the ethical sensitivity of the research, an advisory group of professionals in the field of domestic violence, advocacy bodies, and animal welfare organizations will be created [52]. Experts participating in the advisory group are excluded from the secondary target population. The principal researcher candidate and 2 research advisors will hold meetings via Zoom (Zoom Video Communications) prior to ethics submission and after analysis. The advisory group is sought for their expertise regarding sensitivity, recruitment pathways, research questions, and participation sheets. After the analysis, for advice on recommendations based on the findings from the study.

#### Data Collection Tools

In-depth individual interviews are best suited for “sensitive” populations [52] and web-based options may increase the participant response rate [57]. Hence, the primary target population will be invited to participate in individual semistructured, web-based interviews via Zoom. These interviews are expected to take around 1 hour and will be audio recorded. Focus groups are well suited to discussing beliefs, opinions, and attitudes surrounding programs [52], interventions, and service gaps [58]. Therefore, the secondary target population will be invited to participate in web-based focus groups with audio recordings via Zoom. There will be 4 focus groups nationwide. The focus groups are estimated to last 1 to 2 hours as it is important to allow time during the focus groups for rapport building and voicing opinions [56]. Both types of interviews will be professionally transcribed. Verbal and written consent to participate will be obtained from all participants. All participants will be given the opportunity to review a summary of the transcriptions prior to publication [59].

#### Sample Size

The average number of participants in related qualitative studies identified in the scoping review was 20 (Montgomery et al [5]). The method of the research project is designed to gather in-depth, rich data or high-quality dialogue [60]. Hence, between 12 and 20 participants will be sampled from the primary target group, with the final number of participants being guided by data saturation of main themes, and no new insights or issues are found [61]. The secondary target population will consist of 4 focus groups throughout Australia. When a group consists of high knowledge, a minimum of 4 participants are required to develop accurate information [62] and the probability of identifying themes with 6 participants is higher than 99% [63]. Due to the expertise and knowledge of the participants, there will be a minimum of 4 and a maximum of 8 participants to allow for space and reflection with each group member [56]. The number of participants for the target populations is supported by a recent systematic review of effective sample sizes for saturation in qualitative research [61].

#### Data Analysis

Interpretive work is required to identify meanings and themes from participants’ opinions, perceptions, and experiences to meet the research aims and overall purpose. Thematic analysis will be used to provide a systematic approach to coding and conceptualizing themes [58]. Areas of analysis will include the impacts and outcomes of forced separation, accessibility of services, types of unmet needs, experiences of accessing services, and benefits of existing services. When the analysis of each step is completed, a critical analysis will be completed to aggregate the data [58] to provide a complete picture of the policy framework [54]. NVivo 12 software (Lumivero) will be used to facilitate the data analysis process.

## Results

A scoping review of forced separation of companion animals in crisis situations has been completed, identifying the research gaps and guiding the research questions and design for the research project. As of August 2023, supporting organizations have commenced the distribution of the research flyers. Expected data collection will be completed between August and October 2023. The results are expected to be published in June 2025.

## Discussion

### Expected Findings

It is expected that the findings will identify the substantial issues experienced by women and pets in domestic violence situations such as psychological distress, grief, loss, and the complexity of decision-making when considering a pet. It is expected that women and pets need to be considered more seriously in Australia and the development of policies and services needs to include the consideration of pets in safety planning, accommodation, and long-term housing as their standard practice.

### Comparisons With Prior Work

The research protocol builds on existing knowledge in the literature. We are unaware of any published national Australian frameworks or models that directly relate to responding to women and pets fleeing domestic violence. Previous literature indicates when women are seeking help to flee from domestic violence, the risk of safety increases for both women and their pets. In addition, the pet may be used as a coercive control measure, risking further abuse for both the woman and the animal [18]. The evidence indicates it is vital to address the increased risks to safety when fleeing domestic violence. The prospective data collection of service providers and women using domestic violence and animal welfare services in Australia, as we propose in this study, enables further understanding and development of an Australian framework that is embedded by those with lived experiences to improve outcomes.

### Strengths and Limitations

Limitations include the small sample sizes that will not be generalizable to the wider populations, and the exclusion of non–English-speaking populations limits the ability of the research to understand the special needs of the linguistic and cultural populations [55]. The primary target population is recruited from service providers and is considered safe to participate. This is a limitation for women and pets in situations that did not seek formal service provision, had stayed in the relationship, or were not safe from abuse. Bias is more likely to occur in qualitative research than in quantitative methods, resulting in difficulty reaching true objectivity [59]. However, the strength of the qualitative design allows for flexibility and sensitivity in language, trust, rapport building, exploration of experiences, and collaboration within the community [58] and is appropriate for the study’s aims.

### Conclusions

A research project guided by a conceptual framework informed by the findings of the scoping review confirms 4 key studies required to better understand the strengths, needs, and gaps of existing policy and support services for women and pets fleeing domestic violence, and the impacts of forced separation from companion animals. Ultimately, the project will develop an Australian national framework that will develop and provide more relevant guidance for supporting women and their pets fleeing domestic violence situations to improve outcomes for both women and their companion animals in Australia.

## Appendix

Multimedia Appendix 1 Description of the scoping review articles by separation event.

## Abbreviations

PRISMA: Preferred Reporting Items for Systematic Reviews and Meta-Analyses

## Data Availability

Data sharing is not applicable to this article as no data sets were generated or analyzed during this study.
