# Supplementary material for: The Impact of Forced Separations Between Women and Their Pets in Domestic Violence Situations and the Effectiveness of Crisis Response: Protocol for a Conceptual Framework
Source: JMIR Res Protoc. 2024 Jan 25;13:e52067. doi: 10.2196/52067 (PMC10853852; doi:10.2196/52067)
Supplement: Multimedia Appendix 1 [file resprot_v13i1e52067_app1.docx]

Table S1. Description of the 42 articles by separation event: Domestic violence, Health, Homelessness, Housing, and Natural disaster.

| Reference No. | Author/Year | Title | Focus of research | | Methodology | | | Recruitment/target Population |
| --- | --- | --- | --- | --- | --- | --- | --- | --- |
| Domestic violence articles | | | | | | | | |
| 18 | Allen et al., 2006 | Domestic violence and the abuse of pets: Researching the link and its implications in Ireland | The link between animal abuse and domestic violence | | Qualitative methods,  Questionnaire completed in written form-staff supported completion if required.  Analysis not stated. | | | Women Emergency shelters in Ireland. *n* = 23 |
| 20 | Ascione, 1998 | Battered women's reports of their partners' and their children's cruelty to animals | The prevalence of animal abuse and domestic violence | | Quantitative methods, surveys   - Battered partner shelter survey   Descriptive strategy | | | Women in Emergency shelter U.S. Staff administered |
| 23 | Ascione et al., 2007 | Battered pets and domestic violence | Domestic violence and animal abuse and children wellbeing | | Quantitative methods,  comparative study.  surveys   - Conflict tactic scales - Battered partner shelter survey - Families and pets survey   Descriptive statistics, cross-tabulations, and parametric and nonparametric analyses ,logistic regressions | | | Convenience sample. Non shelter group from community, shelter group from emergency shelters in U.S. *n* = 221 |
| 6 | Barrett et al., 2020 | Animal maltreatment as risk marker of more frequent and severe forms of intimate partner violence | The relationship between animal abuse, types of domestic violence and abuse severity. | | Quantitative methods, surveys   - Partner treatment of animal Scale - Revised conflict tactic scale - Checklist of controlling behaviour   Anova tests | | | Recruitment from women’s emergency transitional shelters in Canada. Staff administered surveys.  Purposive sampling. *n* = 86 |
| 8 | Barrett et al., 2018 | Help-seeking among abused women with pets: Evidence from a Canadian sample | The role of animal abuse and women’s help seeking or deterrence of help seeking (Main focus on role of animal abuse) | | Quantitative methods, surveys   - Partner treatment of animals’ scale - Revised conflict tactic scale - Additional Likert scale questions.   Anova tests | | | Recruitment from women’s emergency transitional shelters in Canada. Staff administered surveys.  Purposive sampling. *n* = 86 |
| 24 | Carlisle-Frank et al., 2004 | Selective battering of the family pet | Beliefs, perceptions, Behaviors of abusers; companion animals as property, a scapegoat, stress/environment | | Quantitative methods,  comparative study of pet abusers and non-pet abusers.   - Sentient being scale - Hassle stressors scale - Unrealistic expectations scale   Analysis- Chi square  Impaired t-tests two tailed  Regression model | | | Recruited from women’s domestic violence shelters.  Written questionnaires provided by staff in U.S. *n* = 34 |
| 7 | Collins et al., 2018 | A template analysis of intimate partner violence survivors’ experiences of animal maltreatment: Implications for safety planning and intervention | The relationship of animal abuse in women and children and barriers to leave | | Qualitative methods, (Part of a mixed method study)   - Pet treatment survey   Semi structured survey completed in written form. (No verbal or audio interviews recorded)  Template analysis | | | Convenience sampling. Recruitment from women’s domestic violence agencies across western U.S. Survey completed by staff writing answers verbatim, or participants completed themselves. Response rate estimated 35.4%. *N*=103 |
| 19 | Faver & Strand, 2003 | To leave or to stay? Battered women’s concern for vulnerable pets | The role of animal abuse and difference in geographical location | | Quantitative methods, surveys   - Domestic violence pet abuse survey   Descriptive statistics, Logistic regression analyses. | | | Two rural and four urban women’s domestic violence shelters in U.S. Estimated response rate 5.68%. *n* = 41 |
| 9 | Fitzgerald et al., 2019 | Animal Maltreatment in the Context of Intimate Partner Violence: A Manifestation of Power and Control? | The connection of animal abuse and domestic violence | | Quantitative methods, surveys   - Partners treatment of animal’s scale - Revised conflict tactic scale   Analysis correlation matrix. | | | Women’s emergency shelters across Canada. *n* = 86 |
| 25 | Flynn, 2000 | Woman’s best friend: Pet abuse and the role of companion animals in the lives of battered women | The role of pets in domestic violence (relationship) | | Quantitative methods,  Likert surveys  Analysis not stated | | | Recruitment women’s emergency shelter in Canada. Staff administered. *n* = 111 |
| 10 | Hardesty et al., 2013 | Coercive control and abused women's decisions about their pets when seeking shelter | Connection of animal abuse, domestic violence and decision making | | Qualitative methods,  Face to face interviews.  Analysis grounded theory. | | | Recruitment from women’s domestic violence shelter in U.S. Staff identified participants. Researchers completed interview. *n* = 19 |
| 26 | Hartman et al., 2018 | Intimate Partner Violence and Animal Abuse in an Immigrant-Rich Sample of Mother-Child Dyads Recruited from Domestic Violence Programs | Connection of animal abuse and domestic violence | | Quantitative methods, surveys   - Revised conflict tactic scale - Pet treatment survey - Cruelty to animals’ inventory   Logistics regression analysis | | | Recruitment from women’s domestic violence shelters in U.S. Staff selected participants and administered surveys.  *n* = 291 |
| 27 | Simmons & Lehmann, 2007 | Exploring the link between pet abuse and controlling behaviours in violent relationships | Connection of animal abuse and domestic violence | | Quantitative methods, surveys   - Likert scale - Checklist of controlling behaviours   Descriptive analysis, Chi-Square, two tailed t-test | | | Recruitment through a women’s urban domestic violence shelter. *n* = 1283 |
| 21 | Strand & Faver, 2005 | Battered women's concern for their pets: a closer look | Connection of animal abuse and domestic violence (decision making) | | Mixed methods  Quantitative survey for participants   - Pet abuse survey   Qualitative interview with Domestic violence worker in rural shelter.  Frequency distributions, descriptive statistics. Contingency tables, Chi-square analyses, and the Phi-coefficient. | | | Recruitment through two (rural & urban) women’s domestic violence shelters in U.S. Response rate 38% urban, 58% rural. *n* = 51 |
| 3 | Taylor et al., 2018 | People of diverse genders and/or sexualities caring for and protecting animals’ companions in the context of domestic violence | The role of companion animals in domestic violence through the lens of diverse genders | | Qualitative methods (Part of a mixed method study)  Online survey, three open ended questions from a 42-item questionnaire focusing on experience for qualitative approach. Thematic analysis  (Scales below are from larger quant study)   - Pet attitude scale - Liking people scale - Kessler psychological distress scale - Multi-dimensional scale of perceived social support. | | | Recruitment through social media and emails through organization in Australia and U.K. Estimated response rate 27.24%. *n* = 137 |
| 4 | Tiplady et al., 2012 | Intimate partner violence and companion animal welfare | Effect of domestic violence on the companion animal | | Quantitative methods,  survey questionnaire.  Telephone interviews. | | | Recruitment from the Australian public by advertising posters, radio, animal welfare and IPV victim support websites and newspaper articles. *n* = 26 |
| 11 | Tiplady et al., 2018 | The animals are all I have Domestic Violence, Companion Animals, and Veterinarians | Issues of Domestic violence, animal abuse and vet care | | Qualitative methods,  Semi-structured interviews | | | Recruitment through domestic violence crisis line or stayed at a QLD-Australian women’s domestic violence shelter. Participants selected by staff at refuge and directed to researcher. *n* = 13 |
| 28 | Volant et al., 2008 | The relationship between domestic violence and animal abuse | Domestic violence, animal abuse rural/urban (Victoria-Australia) | | Quantitative methods, comparative study. Administered via telephone.  Quantitative questionnaire.  Chi-square, t-test analysis | | | Recruitment through women’s domestic violence shelters in Victoria-Australia and non-domestic violence group from neighbour-hood community houses. *n* = 204 |
| Health Articles | | | | | | | | |
| 29 | Applebaum et al., 2020 | How pets’ factor into healthcare decisions for COVID-19: A One Health perspective | | Decision making in the context of health care (Covid-19) and pets | | Mixed methods-  Online questionnaire survey and   - Multidimensional scale of perceived social support.   Convergent comparison design. Multinomial logistic regression models, grounded theory | Non-probability sampling. Social media interest groups. Adult pet owners in the US. *n* = 2772 | |
| 30 | Canady & Sansone, 2019 | Health care decisions and delay of treatment in companion animal owners | | Decision making in the context of accessing health care and pets | | Quantitative methods, surveys   - Interpersonal support evaluation list - Monash dog ownership relationship scale   comparison groups, linear regression analysis | U.S community sample recruited via Amazon mechanical Turk. Companion animal owners.  *n* = 148 (questionnaire)  *n* = 263 (comparison groups) | |
| Homelessness Articles | | | | | | | | |
| 31 | Cleary et al., 2021 | The Unbreakable Bond: The Mental Health Benefits and Challenges of Pet Ownership for People Experiencing Homelessness | | To explore the experiences of pet owners  who are or were homeless | | Qualitative methods,  face to face interviews.  Narrative thematic analysis | Homeless adults recruited through homeless services in Sydney Australia. *n* = 2 | |
| 32 | Cronley et al., 2009 | Homeless people who are animal caretakers: a comparative study | | Differences in characteristics between homeless with animals and homeless without animals declining housing out of concern for their animals. | | Quantitative methods, nonprobability purposive sampling.  Cross tabulations, bivariate correlations | Data collected from client's self-reports collected from the Homeless Management Information System in the U.S. Homeless people who report caring for animals with homeless people who do not report caring for animals. *n* = 4,100 | |
| 33 | Kidd & Kidd, 1994 | Benefits and liabilities of pets for the homeless | | To examine the attachment and correlation of people owning pets in childhood and level of attachment in adult hood | | Qualitative methods,  face to face interviews. | Recruitment from soup kitchens and outreach centres in the U.S. Homeless adults. *n* = 105 | |
| 34 | Labrecque & Walsh, 2011 | Homeless women's voices on incorporating companion animals into shelter services | | To examine the nature of animal caretaking among female, homeless shelter residents | | Qualitative methods, face to face interviews. Phenomenological/ content analysis | Women recruited through urban homeless shelters in Canada.  *n* = 51 | |
| 35 | Scanlon et al., 2021 | Homeless People and Their Dogs: Exploring the Nature and Impact of the Human Companion Animal Bond | | To explore the nature of the Human– Companion Animal Bond between UK homeless owners and their dogs | | Qualitative methods,  semi-structured interviews. Thematic analysis | Homeless or risk of homeless participants recruited through a U.K housing service. *n* = 20 | |
| 36 | Singer et al., 1995 | Dilemmas associated with rehousing homeless people who have companion animals | | To assess the nature and consequences of human/animal relationships amongst the homeless. | | Quantitative surveys.   - Lexington attachment to pets’ scale. - Beck hopelessness scale   Statistical analysis, t-test. | Homeless people seeking a vet clinic for homeless pet owners in the U.S. *n* = 66 | |
| 37 | Slatter et al., 2012 | Homelessness and companion animals: More than just a pet? | | To explore the extent to which homelessness impacts on the ability to have animal companions. | | Qualitative methods,  semi-structured interviews. Descriptive analysis | Clients of a homeless health Outreach Team in Australia.  *n* = 26 | |
| 22 | Wusinich et al., 2019 | If you're gonna help me, help me: Barriers to housing among unsheltered homeless adults | | To examine barriers accessing housing, services and experiences surviving on the street | | Qualitative interviews, Critical realist framework, thematic analysis. | Stratified random sampling through unsheltered homeless participants in the U.S. *n* = 43 | |
| Housing Articles | | | | | | | | |
| 38 | Shore et al., 2003 | Moving as a reason for pet relinquishment: a closer look | | The relationship between bonding and relinquishment for moving | | Qualitative open-ended questionnaires-telephone surveys.   - human-animal bond scale (exact scale not stated) | Recruitment from a human society (charity organization) in the U.S. Response rate 68.40%. *n* = 57 | |
| Natural Disaster Articles | | | | | | | | |
| 39 | Brackenridge et al., 2012 | Dimensions of the human-animal bond and evacuation decisions among pet owners during Hurricane Ike | | To examine pet owner evacuation in a post-Katrina PETS Act environment | | Quantitative surveys   - Lexington attachment to pets scale - Miller-Rada commitment to pets scale   Descriptive and  bi-variate statistics and logistic regressions. | A self-survey mailed to post codes of pet-owning residents in Harris County in the U.S  that had been under a mandatory evacuation order. *n* = 120 | |
| 40 | Coombs et al., 2015 | Did dog ownership influence perceptions of adult health and wellbeing during and following the Canterbury earthquakes? A qualitative study | | Health perceptions of healthy adults during and post-earthquake | | Qualitative methods, interviews, thematic analysis | Recruitment through purposive sampling with word of mouth from the Christchurch city and townships. Participants were women who owned a dog at the time of the earthquake in New Zealand. *n* = 7 | |
| 41 | Farmer & DeYoung, 2019 | The pets of Hurricane Matthew: Evacuation and sheltering with companion animals | | Companion animals and evacuation decision making-with a focus on pets of Hurricane Mathew | | Mixed Methods-  Open-ended questionnaire,  stated choice surveys, thematic analysis, chi-square analysis | A convenience sample recruited through social media of owners who lived in areas affected by Hurricane Matthew in the U.S.  *n* = 214 | |
| 42 | Heath et al., 2001 | Risk factors for pet evacuation failure after a slow-onset disaster | | Risk factors for pet evacuation failure during a flood | | Quantitative methods, evacuation-based questionnaire | Random telephone survey for pet-owning persons under evacuation notice. *n* = 203 | |
| 43 | Hunt et al., 2008 | Psychological sequelae of pet loss following Hurricane Katrina | | To assess the psychological effects of pet loss post Hurricane Katrina | | Quantitative methods, self-report surveys-online   - Beck depression inventory - PTSD symptom scale self-report - Peri-traumatic dissociative experiences questionnaire - Stanford acute stress reaction questionnaire - Pet attachment questionnaire - Pet bereavement questionnaire   Monova, Anova, Cohen’s d analysis | Recruitment through relevant social media websites in the U.S. *N*=65 | |
| 44 | Lowe et al., 2009 | The impact of pet loss on the perceived social support and psychological distress of hurricane survivors | | Perceived social support and elevated psychological distress post disaster. | | Quantitative methods, Likert surveys.   - Social provisions scale - Kessler-6 scale   Conservation of resources analysis | Recruitment from a New Orleans educational interventions. A sample of low-income African American single mother’s post Hurricane Katrina with pet loss.  *n* = 365 | |
| 45 | Taylor et al., 2015 | The preparedness and evacuation behaviour of pet owners in emergencies and natural disasters | | Pet owner emergency preparedness or anticipated evacuation behaviours in the context of an experienced disaster or emergency | | Quantitative online survey based on evacuation.  Simple descriptive statistics, frequencies, and cross tabulations | Recruitment through social media. Australian Pet owners who experienced a range of natural disasters or emergencies. *n* = 352 | |
| 46 | Thompson et al., 2012 | Pet ownership and the spatial and temporal dimensions of evacuation decisions | | Evacuation decision-making to include or not include pets during Hurricane Gustav | | Quantitative methods. Surveys focused on evacuation completed face to face. | Recruitment through convenience sampling, rest stops along major evacuation routes (Gas stations, convenience stores, hotel), response rate 65%. *n* = 119 | |
| 47 | Thompsons et al., 2017 | Animal Ownership Among Vulnerable Populations in Regional South Australia: Implications for Natural Disaster Preparedness and Resilience | | Preparedness/survival planning and animals for a bushfire emergency. | | Quantitative methods, surveys based on preparedness, perceived risks/responses. Univariate descriptive statistics, chi-square, independent t tests analysis | Recruitment online from affected areas in South Australia and firefighting agency with animal owners threatened by bushfire in January 2014. *n* = 606 | |
| 48 | Trigg et al., 2016 | A moveable beast: Subjective influence of human-animal relationships on risk perception, and risk behaviour during bushfire threat | | Examination of the human-animal connections and risk perception and behaviour in companion animals at times of bushfire. | | Qualitative methods, semi-structured face to face interviews at participants residence  Critical incident framework and thematic analysis | Recruitment through social media and community notices e.g., veterinary clinics. South Australian residents in bushfire-affected area. *n* = 25 | |
| 49 | Trigg et al., 2019 | Archetyping relationships with companion animals to understand disaster risk-taking propensity | | To test an archetypal profiling approach to understanding animal-related, disaster risk-taking, motives of pet–owners | | Quantitative methods surveys below:   - Pet attachment questionnaire - Possession attachment scale - Incorporation into the extended self-scale - Emotional significance scale - Anthropomorphism Scale - Companion Animal Self object Questionnaire - Mini-International Personality Item Pool scales - Environmental Attitude Orientation Scale - Risk Propensity Scale - Pet–Owner Risk Propensity Scale - a ‘moral dilemma’ vignette - A social-desirability bias scale   Exploratory two-step cluster analysis | Recruitment of Australian pet owners through social media online websites and Qualtrics, Provo, UT. Response rate 33%.  *n* = 437 | |
| 50 | Yamazaki, 2015 | A survey of companion animal owners affected by the East Japan Great Earthquake in Iwate and Fukushima Prefectures, Japan | | To explore preparedness, evacuation and required social support | | Quantitative methods, self-administered surveys   - Questionnaire regarding disasters and pets - Pet attachment scale - Disaster Preparedness Scale - Utilization of Support Scale - Need for Support Scale   Chi-square, Pearson’s product moment correlation, Spearman’s rank correlation, and t-tests | Recruitment handed out in person at veterinary hospitals in Japan and/or telephone or posted. Pet owners affected by the disasters of Fukushima and earthquake. Response rate 70.5%. *n* = 289 | |
| 51 | Zottarelli, 2010 | Broken bond: An exploration of human factors associated with companion animal loss during Hurricane Katrina | | To explore pre-existing characteristics of disaster vulnerability of people who experienced pet loss, and evacuation behaviours and pet loss/trauma | | Quantitative methods, telephone interviews.  Descriptive, bivariate, and multivariate analyses | Random sample of Hurricane Katrina survivors selected through Gallup organization from a U.S database who sought assistance from the American red cross and affiliated organizations post Hurricane Katrina. Response rate 90%. *n* = 1510 | |
